# Supplementary material for: Phosphoproteomics reveals rewiring of the insulin signaling network and multi-nodal defects in insulin resistance
Source: Nat Commun. 2023 Feb 18;14:923. doi: 10.1038/s41467-023-36549-2 (PMC9938909; doi:10.1038/s41467-023-36549-2)
Supplement: Supplementary file 1 — Supplementary Information [file 41467_2023_36549_MOESM1_ESM.pdf]

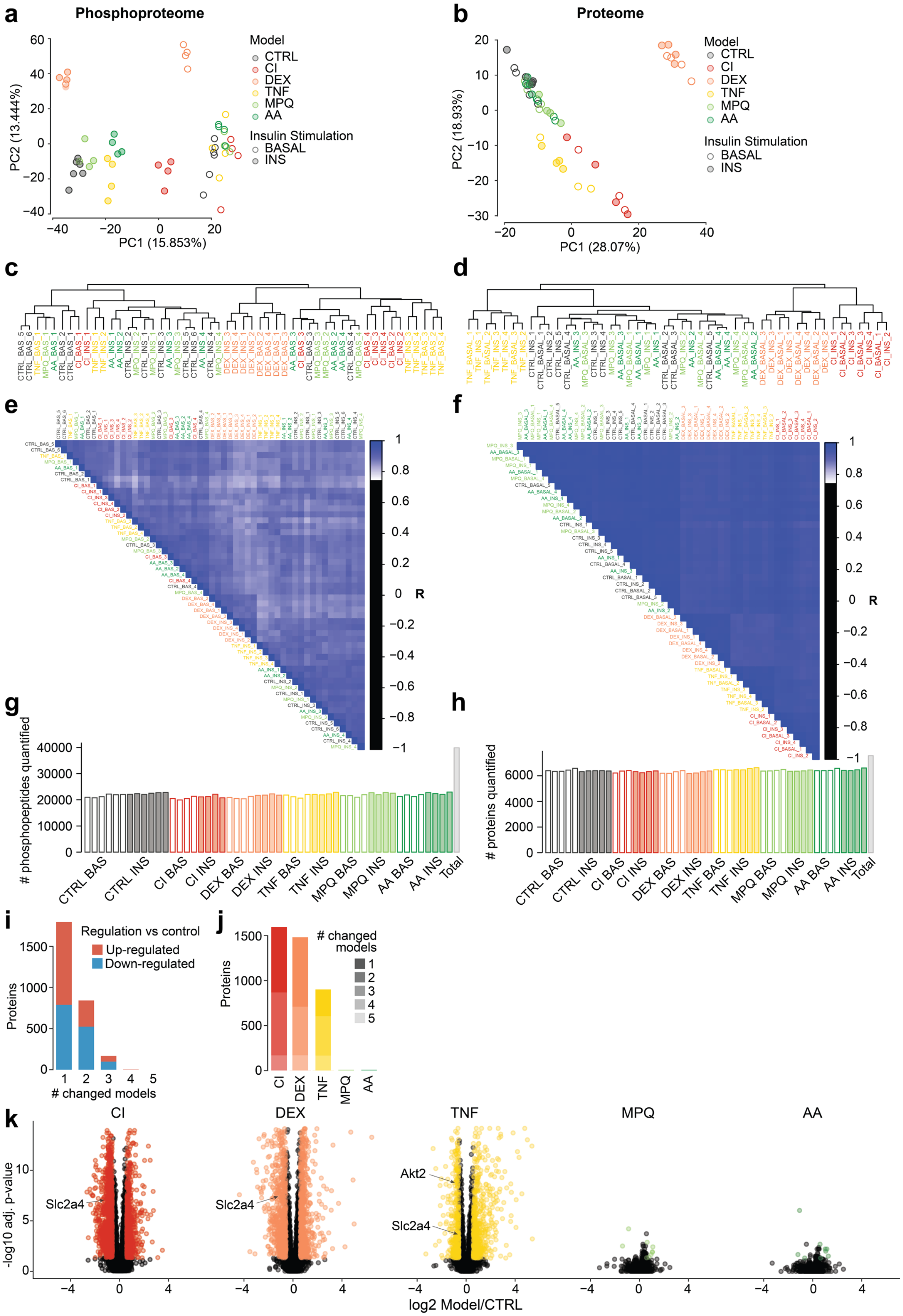

a
Phosphoproteome
Model
CTRL
CI
DEX
TNF
MPQ
AA
Insulin Stimulation
BASAL
INS
PC2 (13.444%)
PC1 (15.853%)
b
Proteome
Model
CTRL
CI
DEX
TNF
MPQ
AA
Insulin Stimulation
BASAL
INS
PC2 (18.93%)
PC1 (28.07%)
c
d
e
R
f
R
g
phosphopeptides quantified
CTRL BAS
CTRL INS
CI BAS
CI INS
DEX BAS
DEX INS
TNF BAS
TNF INS
MPQ BAS
MPQ INS
AA BAS
AA INS
Total
h
proteins quantified
CTRL BAS
CTRL INS
CI BAS
CI INS
DEX BAS
DEX INS
TNF BAS
TNF INS
MPQ BAS
MPQ INS
AA BAS
AA INS
Total
i
Regulation vs control
Up-regulated
Down-regulated
Proteins
changed models
j
changed models
Proteins
CI
DEX
TNF
MPQ
AA
k
CI
DEX
TNF
MPQ
AA
Slc2a4
Slc2a4
Akt2
Slc2a4
-log10 adj. p-value
log2 Model/CTRL

Supplementary Fig. 1 | Analysis of the proteome and phosphoproteome in insulin resistant adipocytes (related to Fig. 1)

(**a-b**) PCA was performed on the 3T3-L1 insulin resistance (a) phosphoproteome or (b) proteome using the base R "prcomp" function. The first two principal components (PC1 and PC2) are plotted for each replicate, and the percentage contribution of these principal components to total variance is indicated. (**c-d**) Hierarchical clustering of the (c) phosphoproteome or (d) proteome using the base R functions "dist" and "hclust". (**e-f**) Pearson's correlation between (e) phosphoproteome or (f) proteome replicates. (**g-h**) Number of quantified phosphopeptides/proteins in each replicate and in total for the (g) phosphoproteome or (h) proteome. (**i-j**) Proteins up- or down-regulated in insulin resistant cells compared to control cells. (i) x-axis indicates the maximum number of models in which each protein is changed. (j) Colour saturation indicates whether proteins are changed only in the indicated model (1 model, most saturated) or are also changed in the same direction in other models (2, 3, 4, 5 models, decreasing saturation). (**k**) Volcano plots showing proteomic changes in each insulin resistance model compared to control cells (Dunnett's post-hoc tests following one-way ANOVA, p-values adjusted by the Benjamini-Hochberg procedure). Significantly altered proteins are coloured, and select proteins are labeled.

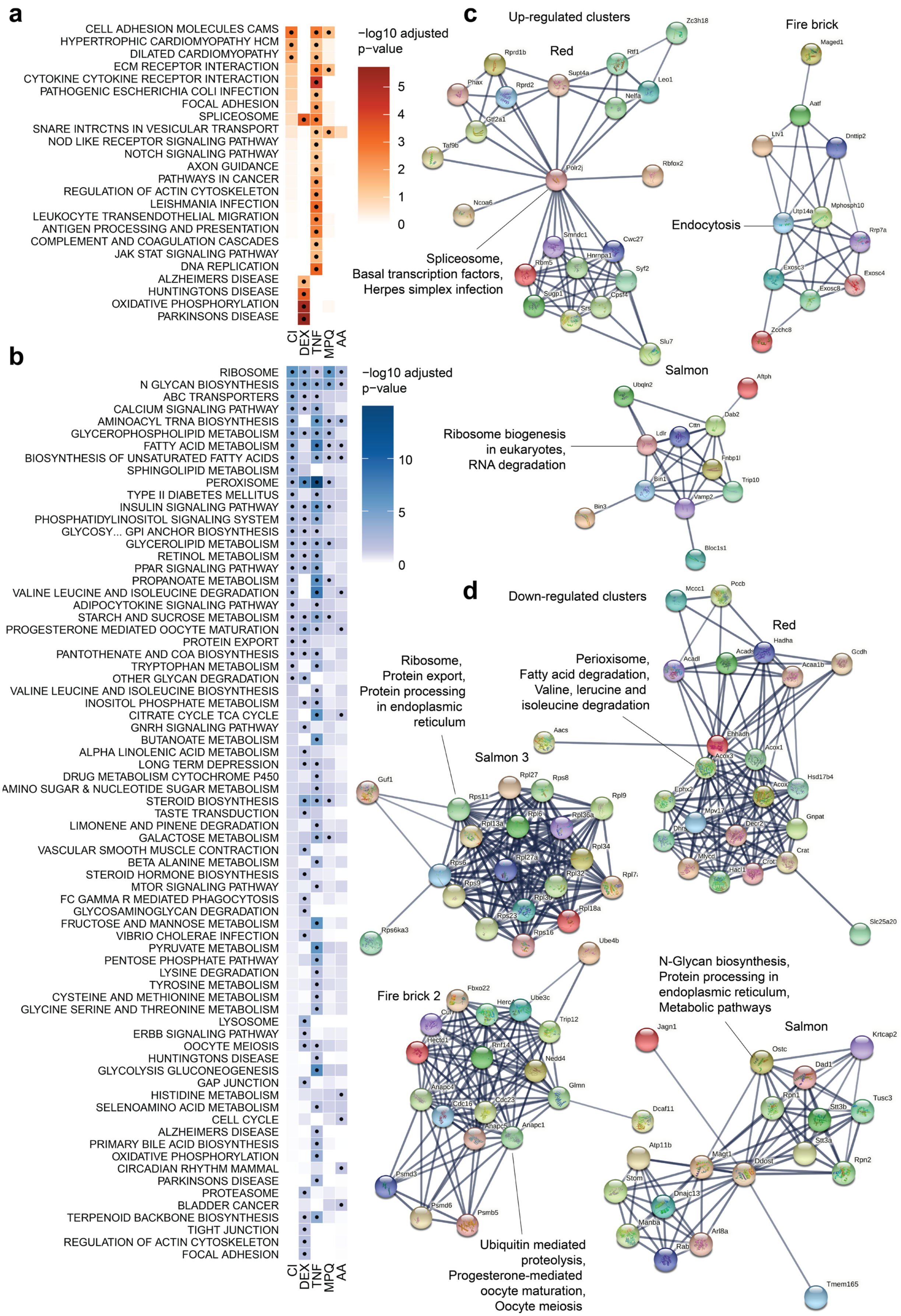
a
CELL ADHESION MOLECULES CAMS
HYPERTROPHIC CARDIOMYOPATHY HCM
DILATED CARDIOMYOPATHY
ECM RECEPTOR INTERACTION
CYTOKINE CYTOKINE RECEPTOR INTERACTION
PATHOGENIC ESCHERICHIA COLI INFECTION
FOCAL ADHESION
SPLICEOSOME
SNARE INTRCTNS IN VESICULAR TRANSPORT
NOD LIKE RECEPTOR SIGNALING PATHWAY
NOTCH SIGNALING PATHWAY
AXON GUIDANCE
PATHWAYS IN CANCER
REGULATION OF ACTIN CYTOSKELETON
LEISHMANIA INFECTION
LEUKOCYTE TRANSENDOTHELIAL MIGRATION
ANTIGEN PROCESSING AND PRESENTATION
COMPLEMENT AND COAGULATION CASCADES
JAK STAT SIGNALING PATHWAY
DNA REPLICATION
ALZHEIMERS DISEASE
HUNTINGTONS DISEASE
OXIDATIVE PHOSPHORYLATION
PARKINSONS DISEASE
CI
DEX
TNF
MPQ
AA
−log10 adjusted p−value
5
4
3
2
1
0
b
RIBOSOME
N GLYCAN BIOSYNTHESIS
ABC TRANSPORTERS
CALCIUM SIGNALING PATHWAY
AMINOACYL TRNA BIOSYNTHESIS
GLYCEROPHOSPHOLIPID METABOLISM
FATTY ACID METABOLISM
BIOSYNTHESIS OF UNSATURATED FATTY ACIDS
SPHINGOLIPID METABOLISM
PEROXISOME
TYPE II DIABETES MELLITUS
INSULIN SIGNALING PATHWAY
PHOSPHATIDYLINOSITOL SIGNALING SYSTEM
GLYCOSY... GPI ANCHOR BIOSYNTHESIS
GLYCEROLIPID METABOLISM
RETINOL METABOLISM
PPAR SIGNALING PATHWAY
PROPANOATE METABOLISM
VALINE LEUCINE AND ISOLEUCINE DEGRADATION
ADIPOCYTOKINE SIGNALING PATHWAY
STARCH AND SUCROSE METABOLISM
PROGESTERONE MEDIATED OOCYTE MATURATION
PROTEIN EXPORT
PANTOTHENATE AND COA BIOSYNTHESIS
TRYPTOPHAN METABOLISM
OTHER GLYCAN DEGRADATION
VALINE LEUCINE AND ISOLEUCINE BIOSYNTHESIS
INOSITOL PHOSPHATE METABOLISM
CITRATE CYCLE TCA CYCLE
GNRH SIGNALING PATHWAY
BUTANOATE METABOLISM
ALPHA LINOLENIC ACID METABOLISM
LONG TERM DEPRESSION
DRUG METABOLISM CYTOCHROME P450
AMINO SUGAR & NUCLEOTIDE SUGAR METABOLISM
STEROID BIOSYNTHESIS
TASTE TRANSDUCTION
LIMONENE AND PINENE DEGRADATION
GALACTOSE METABOLISM
VASCULAR SMOOTH MUSCLE CONTRACTION
BETA ALANINE METABOLISM
STEROID HORMONE BIOSYNTHESIS
MTOR SIGNALING PATHWAY
FC GAMMA R MEDIATED PHAGOCYTOSIS
GLYCOSAMINOGLYCAN DEGRADATION
FRUCTOSE AND MANNOSE METABOLISM
VIBRIO CHOLERAE INFECTION
PYRUVATE METABOLISM
PENTOSE PHOSPHATE PATHWAY
LYSINE DEGRADATION
TYROSINE METABOLISM
CYSTEINE AND METHIONINE METABOLISM
GLYCINE SERINE AND THREONINE METABOLISM
LYSOSOME
ERBB SIGNALING PATHWAY
OOCYTE MEIOSIS
HUNTINGTONS DISEASE
GLYCOLYSIS GLUCONEOGENESIS
GAP JUNCTION
HISTIDINE METABOLISM
SELENOAMINO ACID METABOLISM
CELL CYCLE
ALZHEIMERS DISEASE
PRIMARY BILE ACID BIOSYNTHESIS
OXIDATIVE PHOSPHORYLATION
CIRCADIAN RHYTHM MAMMAL
PARKINSONS DISEASE
PROTEASOME
BLADDER CANCER
TERPENOID BACKBONE BIOSYNTHESIS
TIGHT JUNCTION
REGULATION OF ACTIN CYTOSKELETON
FOCAL ADHESION
CI
DEX
TNF
MPQ
AA
−log10 adjusted p−value
10
5
0
c
Up-regulated clusters
Red
Spliceosome,
Basal transcription factors,
Herpes simplex infection
Fire brick
Endocytosis
Salmon
Ribosome biogenesis
in eukaryotes,
RNA degradation
d
Down-regulated clusters
Red
Perioxisome,
Fatty acid degradation,
Valine, lerucine and
isoleucine degradation
Ribosome,
Protein export,
Protein processing
in endoplasmic
reticulum
Salmon 3
Fire brick 2
Ubiquitin mediated
proteolysis,
Progesterone-mediated
oocyte maturation,
Oocyte meiosis
N-Glycan biosynthesis,
Protein processing in
endoplasmic reticulum,
Metabolic pathways
Salmon

Supplementary Fig. 2 | Pathway and network analysis implicates functional protein modules in insulin resistance (related to Fig. 1)

(**a-b**) KEGG pathway enrichment was performed in each insulin resistance model by one-way gene set test ("geneSetTest" function from the R package "Limma" (version: 3.14)) using log2 Model/Control fold change values and the KEGG pathways, followed by Benjamini-Hochberg p-value adjustment. (a) Pathways that are up-regulated relative to control cells. (b) Pathways that are down-regulated relative to control cells. Dots indicate significantly regulated pathways (Benjamini-Hochberg-adjusted p-value < 0.05). (**c-d**) Markov clustering was performed on the STRING functional networks of (c) proteins up-regulated or (d) proteins down-regulated in two or more insulin resistance models relative to control cells. All clusters containing at least ten proteins are shown, and the top 3 KEGG pathways significantly enriched in each cluster's genes are indicated. Clusters names (for example Red; Fire brick; Salmon) are shown.

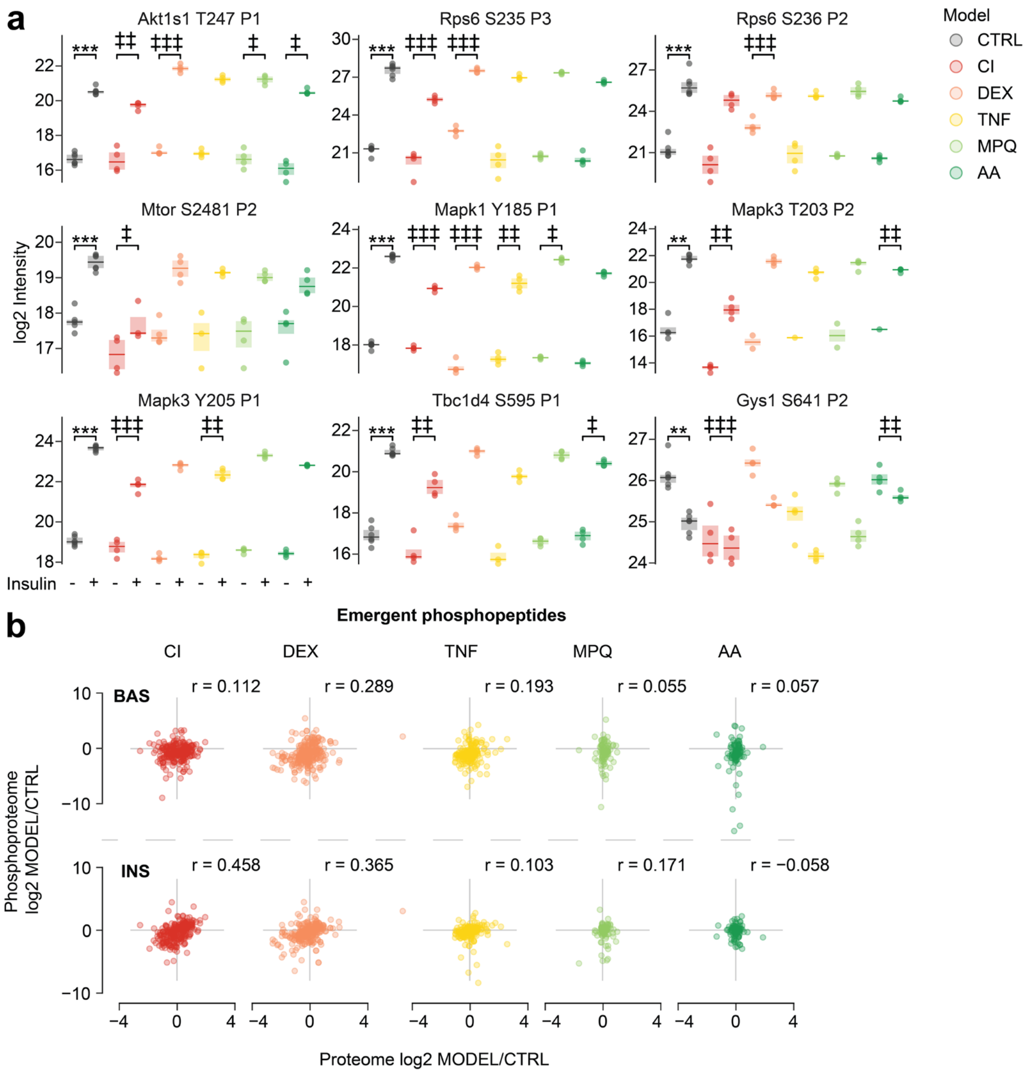

Supplementary Fig. 3 | Canonical and emergent insulin signaling (related to Fig. 2)

(**a**) Select canonical insulin-regulated phosphosites. Two-sided t-tests were performed to compare CTRL BAS to CTRL INS samples following Benjamini-Hochberg p-value adjustment (* with bracket). One-way ANOVAs followed by Dunnett's post-hoc tests with Benjamini-Hochberg p-value adjustment were performed on INS data normalized to BAS median, to compare each insulin resistance model to CTRL (‡ with bracket). "P1/P2/P3" indicates the number of phosphosites on each phosphopeptide. n = 4-6 independent biological replicates. * p-values (left-to-right, top-to-bottom): 7.61e-07, 1.62e-07, 6.65e-05, 0.00057, 1.38e-09, 0.00814, 3.48e-08, 2.75e-05, 0.00236. ‡ p-values (left-to-right, top-to-bottom): 0.00117, 5.03e-05, 0.0104, 0.05, 1.28e-06, 2.01e-05, 3.64e-05, 0.0111, 2.26e-11, 0.000261, 0.00245, 0.021, 0.00196, 0.00912, 4.25e-11, 0.00199, 0.00608, 0.0342, 3.26e-05, 0.00713. (**b**) Correlation of proteome and unstimulated (BAS) or insulin-stimulated (INS) phosphoproteome changes in phosphopeptides emergent in the indicated models. r = pearson's correlation coefficient. */‡: $0.01 < p < 0.05$, **/‡‡: $0.001 < p < 0.01$, ***/‡‡‡: $p < 0.001$.

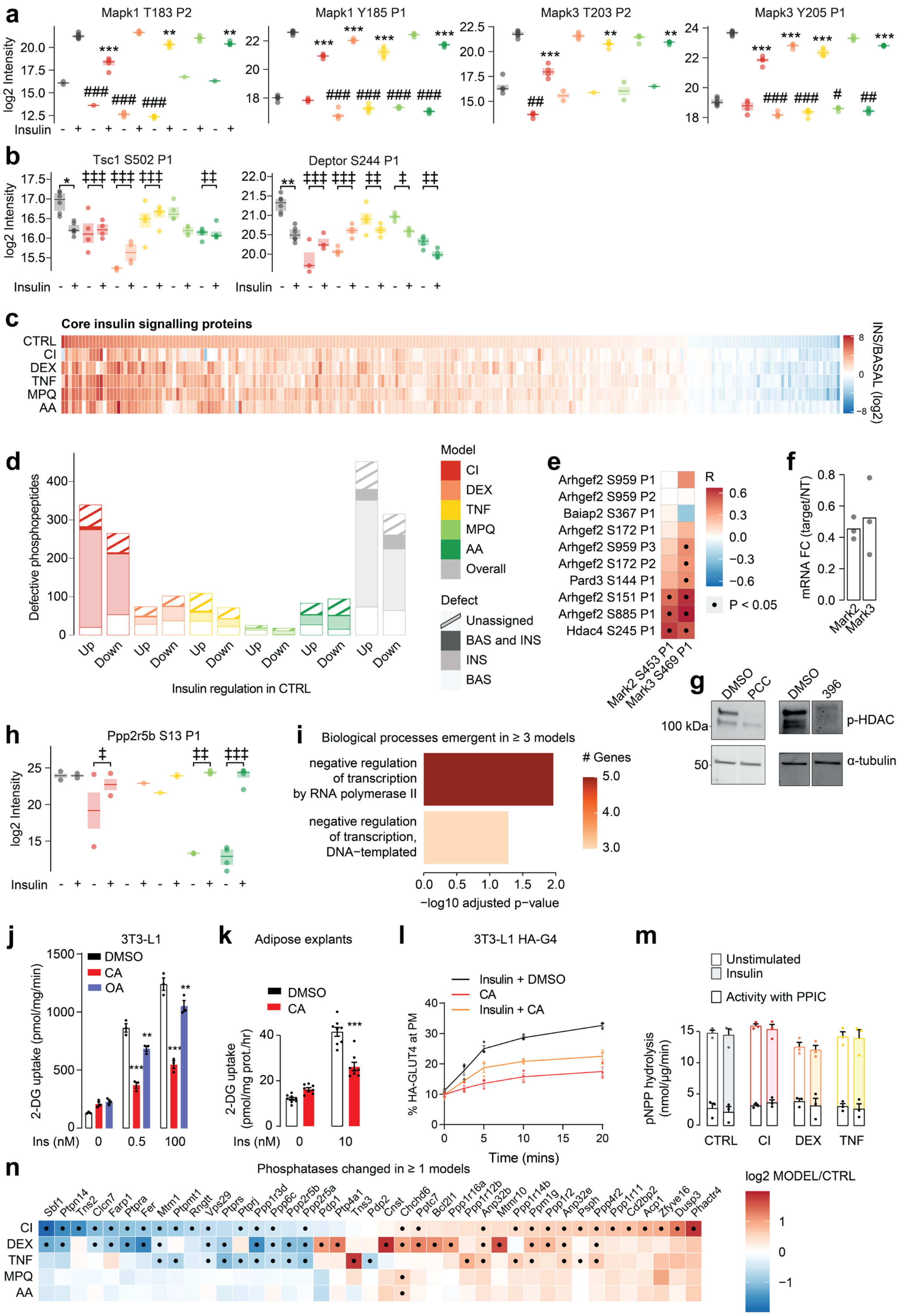

a
Mapk1 T183 P2
Mapk1 Y185 P1
Mapk3 T203 P2
Mapk3 Y205 P1
log2 Intensity
Insulin
b
Tsc1 S502 P1
Deptor S244 P1
log2 Intensity
Insulin
c
Core insulin signalling proteins
CTRL
CI
DEX
TNF
MPQ
AA
INS/BASAL (log2)
d
Defective phosphopeptides
Up
Down
Insulin regulation in CTRL
Model
CI
DEX
TNF
MPQ
AA
Overall
Defect
Unassigned
BAS and INS
INS
BAS
e
Arhgef2 S959 P1
Arhgef2 S959 P2
Baiap2 S367 P1
Arhgef2 S172 P1
Arhgef2 S959 P3
Arhgef2 S172 P2
Pard3 S144 P1
Arhgef2 S151 P1
Arhgef2 S885 P1
Hdac4 S245 P1
Mark2 S453 P1
Mark3 S469 P1
R
P < 0.05
f
mRNA FC (target/NT)
Mark2
Mark3
g
DMSO
PCC
DMSO
396
100 kDa
50
p-HDAC
α-tubulin
h
Ppp2r5b S13 P1
log2 Intensity
Insulin
i
Biological processes emergent in ≥ 3 models
negative regulation of transcription by RNA polymerase II
negative regulation of transcription, DNA−templated
Genes
−log10 adjusted p−value
j
3T3-L1
DMSO
CA
OA
2-DG uptake (pmol/mg/min)
Ins (nM)
k
Adipose explants
DMSO
CA
2-DG uptake (pmol/mg prot./hr)
Ins (nM)
l
3T3-L1 HA-G4
Insulin + DMSO
CA
Insulin + CA
% HA-GLUT4 at PM
Time (mins)
m
Unstimulated
Insulin
Activity with PPIC
pNPP hydrolysis (nmol/µg/min)
CTRL
CI
DEX
TNF
n
Phosphatases changed in ≥ 1 models
log2 MODEL/CTRL
Sbf1 Ptpn14 Tns2 Clcn7 Farp1 Ptpra Fer Mtm1 Ptpmt1 Rngtt Vps29 Ptprs Ptprj Ppp1r3d Ppp6c Ppp2r5b Ppp2r5a Pdp1 Ptp4a1 Tns3 Pdp2 Cnst Chchd6 Pptc7 Bcl2l1 Ppp1r16a Ppp1r12b Anp32b Mtmr10 Ppp1r14b Ppm1g Ppp1r2 Anp32e Psph Ppp4r2 Ppp1r11 Cd2bp2 Acp1 Zfyve16 Dusp3 Phactr4
CI
DEX
TNF
MPQ
AA

Supplementary Fig. 4 | Characterizing defective and emergent insulin signaling (related to Fig. 2)

(**a**) The regulatory phosphosites of Erk1/Mapk3 and Erk2/Mapk1. ANOVAs and Dunnett's post hoc tests were performed on unstimulated (#) or insulin-stimulated (*) phosphoproteome data to compare insulin resistant models to CTRL cells. # p-values (left-to-right): 0.000172, 1.22e-12, 3.08e-07, 5.09e-07, 0.00011, 0.000279, 1.36e-06, 0.00222, 0.000151, 0.000492, 0.0302, 0.00442. * p-values (left-to-right): 7.33e-12, 0.00179, 0.00704, 5.48e-14, 0.000794, 2.21e-12, 1.3e-06, 1.1e-14, 0.0016, 0.00957, 1.11e-16, 5.7e-06, 1.67e-10, 4.37e-06. n = 4-6 independent biological replicates. (**b**) Select phosphosites on canonical insulin signaling proteins that displayed defects in insulin resistance models. Two-sided t-tests were performed to compare CTRL BAS to CTRL INS samples following Benjamini-Hochberg p-value adjustment (* with bracket). One-way ANOVAs followed by Dunnett's post-hoc tests with Benjamini-Hochberg p-value adjustment was performed on INS data normalized to BAS median, to compare each insulin resistance model to CTRL (‡ with bracket). n = 4-6 independent biological replicates. * p-values (left-to-right): 0.0110, 0.00164. ‡ p-values (left-to-right): 0.0000872, 0.0000186, 0.000347, 0.00183, 1.17E-12, 2.17E-12, 0.00314, 0.0270, 0.00675. (**c**) Phosphopeptides on core insulin signaling pathway proteins that were quantified in every condition. (**d**) The distribution of BAS and INS defects among phosphopeptides with defective insulin regulation. For a proportion of defective phosphopeptides defects could not be significantly attributed to BAS or INS (Unassigned). (**e**) Associations between Mark2/3 substrates annotated in PhosphositePlus and the indicated phosphopeptides on Mark2/3 were tested by Pearson's correlation tests without p-value adjustment. (**f**) Fold change of Mark2/3 mRNA in Mark2/3 siRNA-transfected vs non targeting siRNA-transfected (NT) 3T3-L1 adipocytes, measured by RT-qPCR. n=3 biologically independent samples. (**g**) Western blots visualizing phosphorylation of the Mark2/3 substrate pS246/S259/S155 HDAC4/5/7 in 3T3-L1 adipocytes 90 min after incubation in 0.5 μM PCC or 10 μM 396. Lanes removed between DMSO and 396 from right hand blot for clarity. n = 4 independent biological replicates. (**h**) Ppp2r5b S13, a phosphosite that displayed emergence in CI, MPQ, and AA. This site was removed from Fig. 2G to improve the heatmap scale. Statistical tests were performed as in (b). ‡ p-values (left-to-right): 0.0418, 0.00186, 0.0000224. (**i**) GO pathway enrichment on genes containing phosphopeptides that displayed emergent up-regulation (red) or emergent down-regulation in three or more insulin resistance models. Enrichment was assessed by one-way Fisher's exact tests followed by Benjamini-Hochberg p-value adjustment. All pathways that were significant at $p < 0.1$ after Benjamini-Hochberg p-value adjustment are displayed (only biological processes with up-regulated phosphopeptides were significant), and color gradients indicate the number of phospho-emergent genes in each pathway. (**j**) ^{3}H-2DG uptake into 3T3-L1 adipocytes treated with DMSO, calyculin A (CA; 50 nM) or okadaic acid (OA; 1 μM) and insulin at the indicated concentrations for 20 min. Data were analyzed by two-way ANOVA corrected for multiple comparisons (Dunnett's test) to compare between DMSO and CA- or OA-treated cells (*). Error bars = S.E.M. n=3 biologically independent samples. * p-values (left-to-right): < 0.0001,

0.0020, < 0.0001, 0.0013. (**k**) ^{3}H-2DG uptake into epididymal adipose explants treated with DMSO or CA (100 nM) with insulin at the indicated concentrations for 20 min. Data were analyzed by two-way ANOVA corrected for multiple comparisons (Dunnett's test) to compare between DMSO and CA- or OA-treated cells (*). Error bars = S.E.M. n=8 biologically independent samples. * p-value: < 0.0001. (**l**) 3T3-L1 adipocytes overexpressing HA-GLUT4 were treated with insulin (100 nM) and DMSO, CA (50 nM), or insulin and CA. The percentage of HA-GLUT4 at the plasma membrane, as a proportion of total cellular HA-GLUT4, was measured by immunofluorescence at the indicated time points. Data are presented as mean values +/- S.E.M. (**m**) Adipocytes were unstimulated or stimulated with insulin (100 nM) and phosphatase activity was inhibited by phosphatase inhibitor cocktail (PPIC). Total phosphatase activity in 3T3-L1 adipocyte insulin resistant models was measured by hydrolysis of pNPP. Data were analyzed by two-way ANOVA corrected for multiple comparisons (Dunnett's test) to compare between control and insulin resistance models, and unstimulated and insulin-stimulated conditions within models. Error bars = S.E.M. n=3 biologically independent samples. (**n**) Phosphatases that were significantly changed in at least one insulin resistance model compared to control cells. Dots indicate significant differences between models and control. */#/‡: 0.01 < p < 0.05, **/##/‡‡: 0.001 < p < 0.01, ***/###/‡‡‡: p < 0.001.

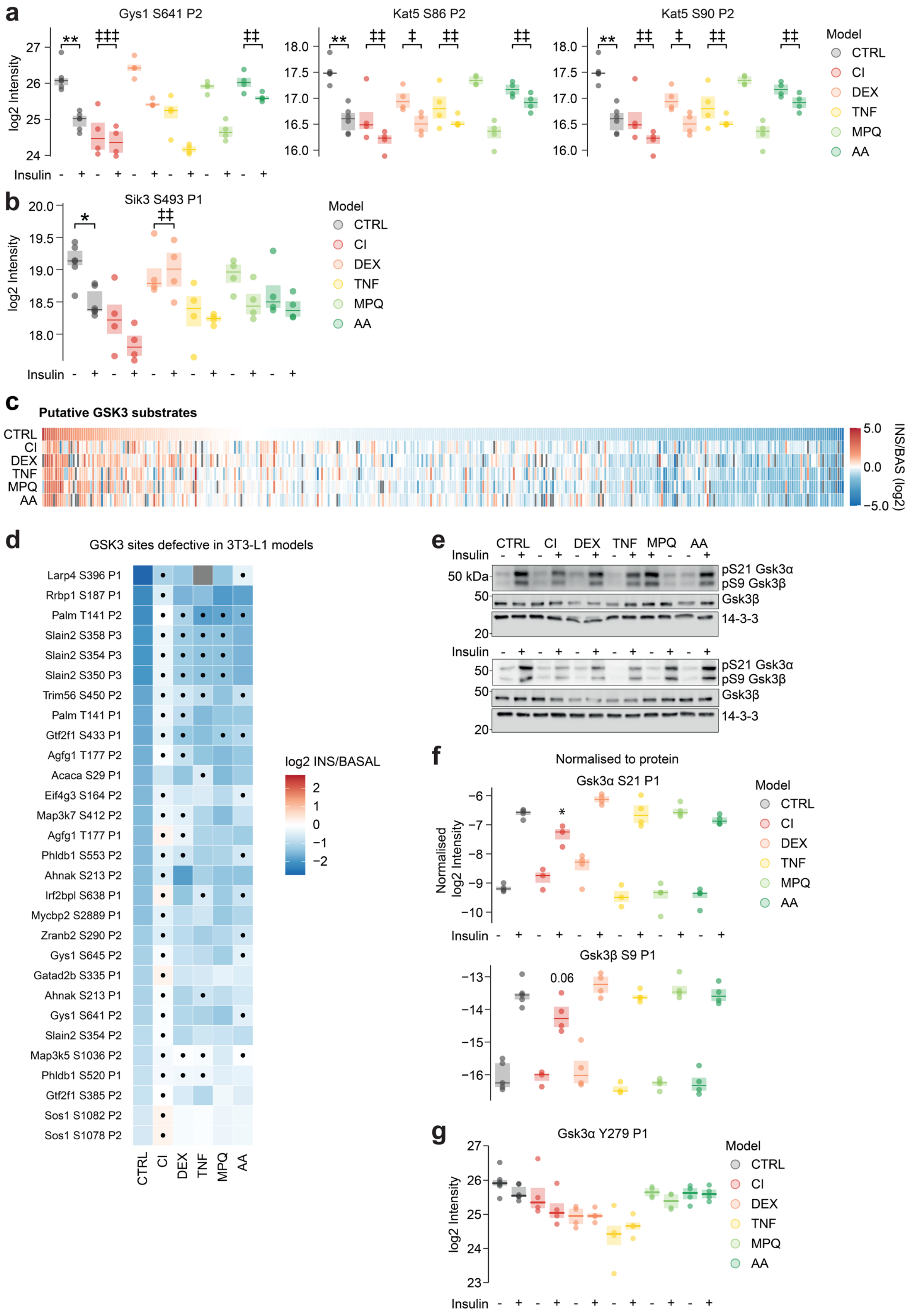
a
Gys1 S641 P2
Kat5 S86 P2
Kat5 S90 P2
log2 Intensity
Insulin
Model
CTRL
CI
DEX
TNF
MPQ
AA
b
Sik3 S493 P1
log2 Intensity
Insulin
Model
CTRL
CI
DEX
TNF
MPQ
AA
c
Putative GSK3 substrates
CTRL
CI
DEX
TNF
MPQ
AA
INS/BAS (log2)
d
GSK3 sites defective in 3T3-L1 models
Larp4 S396 P1
Rrbp1 S187 P1
Palm T141 P2
Slain2 S358 P3
Slain2 S354 P3
Slain2 S350 P3
Trim56 S450 P2
Palm T141 P1
Gtf2f1 S433 P1
Agfg1 T177 P2
Acaca S29 P1
Eif4g3 S164 P2
Map3k7 S412 P2
Agfg1 T177 P1
Phldb1 S553 P2
Ahnak S213 P2
Irf2bpl S638 P1
Mycbp2 S2889 P1
Zranb2 S290 P2
Gys1 S645 P2
Gatad2b S335 P1
Ahnak S213 P1
Gys1 S641 P2
Slain2 S354 P2
Map3k5 S1036 P2
Phldb1 S520 P1
Gtf2f1 S385 P2
Sos1 S1082 P2
Sos1 S1078 P2
CTRL
CI
DEX
TNF
MPQ
AA
log2 INS/BASAL
e
CTRL
CI
DEX
TNF
MPQ
AA
Insulin
50 kDa
pS21 Gsk3α
pS9 Gsk3β
Gsk3β
14-3-3
f
Normalised to protein
Gsk3α S21 P1
Normalised log2 Intensity
Insulin
Gsk3β S9 P1
0.06
Model
CTRL
CI
DEX
TNF
MPQ
AA
g
Gsk3α Y279 P1
log2 Intensity
Insulin
Model
CTRL
CI
DEX
TNF
MPQ
AA

Supplementary Fig. 5 | Impaired GSK3 signaling in insulin resistance (related to Fig. 3)

(**a**) Select Gsk3 substrates that displayed phosphorylation defects in insulin resistance models. Two-sided t-tests were performed to compare CTRL BAS to CTRL INS samples following Benjamini-Hochberg p-value adjustment (* with bracket). One-way ANOVAs followed by Dunnett's post-hoc tests with Benjamini-Hochberg p-value adjustment were performed on INS data normalized to BAS median, to compare each insulin resistance model to CTRL (‡ with bracket). n = 4-6 independent biological replicates. * p-values (left-to-right): 0.00236, 0.00107, 0.00107. ‡ p-values (left-to-right): 3.26e-05, 0.00713, 0.00543, 0.0409, 0.00622, 0.00627, 0.00521, 0.0411, 0.0058, 0.00647. (**b**) Sik3 S493, the only insulin-regulated PKA substrate that was defective in insulin resistance models. Tests were performed as in (a). * p-value: 0.0249. ‡ p-value: 0.00247. (**c**) Putative GSK3 substrates quantified in the 3T3-L1 insulin resistance phosphoproteome. Only phosphopeptides quantified in BAS and INS in at least four models are shown, and missing values are colored gray. (**d**) Gsk3 substrates identified through Gsk3 inhibitor phosphoproteomics that displayed phosphorylation defects in insulin resistance models. Dots indicate that model insulin responses were significantly different to control insulin responses. Missing values are colored gray. (**e**) Visualization of Gsk3α S21, Gsk3β S9, and total Gsk3β by western blotting in two biological replicates. (**f**) Phosphoproteomic intensity of Gsk3α S21 and Gsk3β S9, normalized to the respective total proteins. ANOVAs and two-sided Dunnett's post hoc tests were performed on insulin-stimulated (*) or unstimulated (not significant) phosphoproteome data to compare insulin resistant models to control cells. p-values (top-to-bottom): 0.0137, 0.0602. (**g**) Intensity of Gsk3α Y279. */‡: $0.01 < p < 0.05$, **/‡‡: $0.001 < p < 0.01$, ***/‡‡‡: $p < 0.001$.

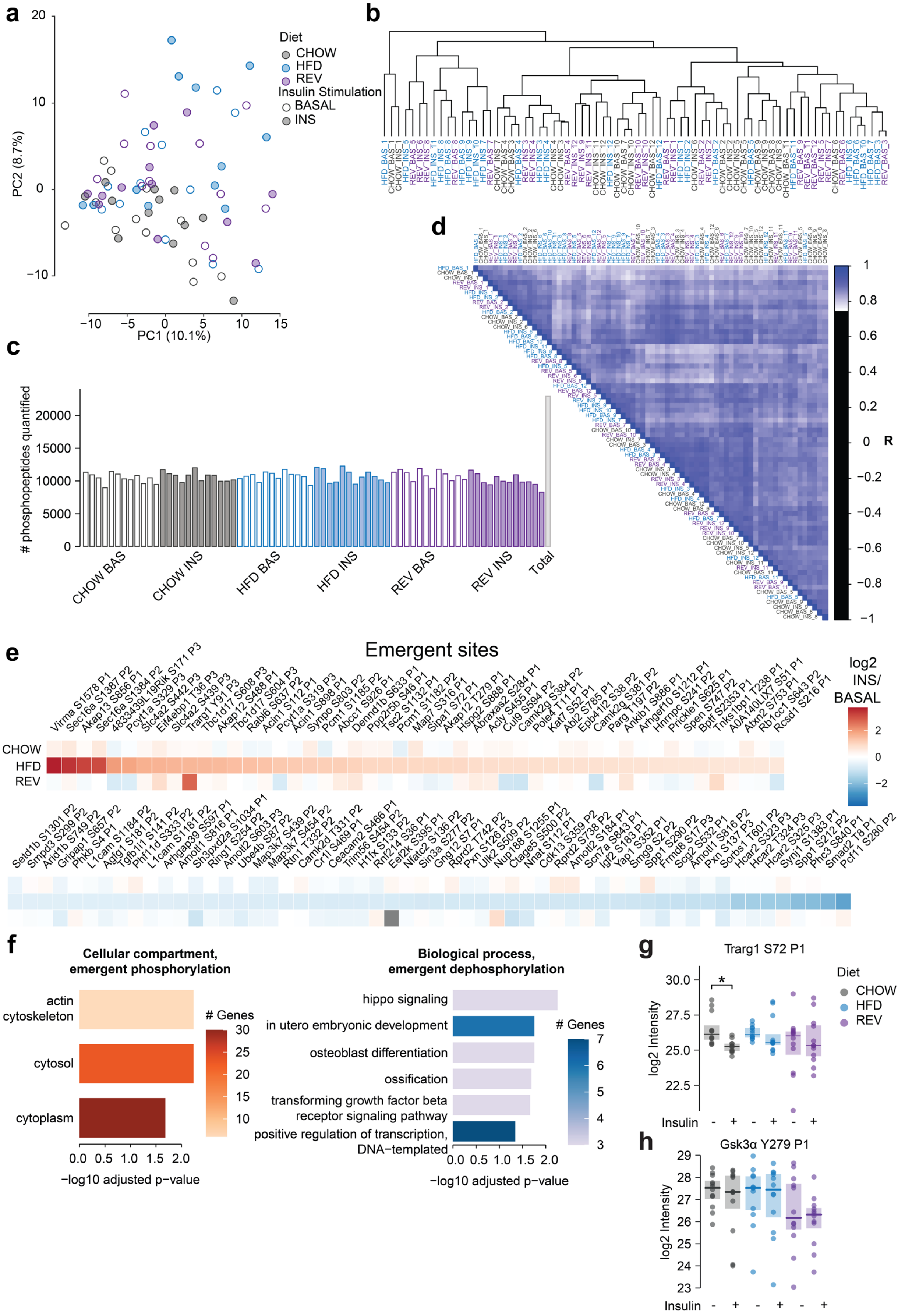
a
PC1 (10.1%)
PC2 (8.7%)
Diet
CHOW
HFD
REV
Insulin Stimulation
BASAL
INS
b
c
phosphopeptides quantified
CHOW BAS
CHOW INS
HFD BAS
HFD INS
REV BAS
REV INS
Total
d
R
e
Emergent sites
log2 INS/ BASAL
CHOW
HFD
REV
Virma S1578 P1
Sec16a S1387 P2
Akap13 S856 P1
Sec16a S1384 P2
4833439L19Rik S171 P3
Pcyt1a S329 P3
Slc4a2 S442 P3
Eif4ebp1 T36 P3
Slc4a2 S439 P3
Trarg1 Y91 P3
Tbc1d17 S608 P3
Akap12 S488 P1
Tbc1d17 S604 P3
Rab6 S637 P2
Acin1 S112 P1
Pcyt1a S319 P3
Acin1 S898 P1
Synpo S803 P2
Pcm1 S1185 P2
Abcc1 S926 P1
Dennd1b S633 P1
Ppp2r5b S46 P1
Tsc2 S1132 P1
Pcm1 S1182 P2
Map7 S316 P1
Sipa1 S72 P1
Akap12 T279 P1
Hspg2 S888 P1
Abraxas2 S284 P1
Acly S455 P1
Cul9 S594 P2
Camk2g S384 P2
Pole4 T11 P1
Kat7 S52 P1
Abi2 S785 P1
Epb41l2 S38 P2
Camk2g S381 P2
Parg T197 P2
Ankib1 S886 P1
Arhgef10 S1212 P1
Hnrnpc S241 P2
Prickle1 S625 P1
Spen S747 P2
Bptf S2353 P1
Tnks1bp1 T238 P1
A0A140LIX7 S51 P1
Atxn2 S753 P1
Rb1cc1 S643 P2
Rcsd1 S216 P1
Setd1b S1301 P2
Smpd3 S299 P2
Arid1b S749 P2
Gripap1 S657 P2
Phkb S4 P1
L1cam S1184 P2
Agfg1 S181 P2
Tgfb1i1 S141 P2
Phf11d S333 P2
L1cam S1181 P2
Arhgap39 S597 P1
Amotl1 S816 P1
Sh3pxd2a S1034 P1
Ring1 S254 P2
Amotl2 S603 P3
Ube4b S87 P2
Map3k7 S439 P2
Map3k7 S454 P2
Rtn1 T332 P2
Camk2d T331 P2
Cr1l S469 P1
Ceacam2 S466 P1
Trim56 S454 P2
H1fx S133 P2
Rnf214 S36 P1
Eef2k S395 P1
Nfatc2 S136 P2
Sin3a S277 P2
Gng12 S7 P1
Rprd2 T742 P2
Pxn S126 P3
Ulk2 S509 P2
Nup188 S1255 P1
Clasp2 S600 P2
Nnat S112 P1
Cdk13 S359 P2
Rprd2 S738 P2
Amotl2 S184 P1
Scn7a S843 P1
Igf2 S183 P1
Yap1 S352 P1
Smg9 S2 P2
Sgo1 S290 P2
Frmd8 S17 P3
Scg2 S532 P1
Amotl1 S816 P2
Pxn S137 P3
Sorbs1 T601 P2
Hcar2 S323 P3
Hcar2 T324 P3
Hcar2 S325 P3
Synj1 S1383 P1
Sgo1 S212 P2
Phc3 S840 P1
Smad2 T8 P1
Pcf11 S280 P2
f
Cellular compartment, emergent phosphorylation
actin cytoskeleton
cytosol
cytoplasm
Genes
−log10 adjusted p−value
Biological process, emergent dephosphorylation
hippo signaling
in utero embryonic development
osteoblast differentiation
ossification
transforming growth factor beta receptor signaling pathway
positive regulation of transcription, DNA−templated
Genes
−log10 adjusted p−value
g
Trarg1 S72 P1
log2 Intensity
Insulin
Diet
CHOW
HFD
REV
h
Gsk3α Y279 P1
log2 Intensity
Insulin

Supplementary Fig. 6 | Altered insulin signaling in insulin resistant adipose tissue (related to Fig. 4) (**a**) PCA was performed on the adipose tissue insulin resistance phosphoproteome using the base R "prcomp" function. The first two principal components (PC1 and PC2) are plotted for each replicate, and the percentage contribution of these principal components to total variance is indicated. (**b**) Hierarchical clustering of the phosphoproteome using the base R functions "dist" and "hclust". (**c**) Number of quantified phosphopeptides in each replicate of the phosphoproteome and in total. (**d**) Pearson's correlation between phosphoproteome replicates. (**e**) Emergent phosphopeptides that were regulated by insulin in HFD mice but not in CHOW mice. Missing values are colored gray. (**f**) GO pathway enrichment on genes containing phosphopeptides with emergent up-regulation (red) or down-regulation (blue) in HFD mice. Enrichment was assessed by one-sided Fisher's exact tests followed by Benjamini-Hochberg p-value adjustment. All pathways that were significant after Benjamini-Hochberg p-value adjustment are displayed, and color gradients indicate the number of phospho-emergent genes in each pathway. (**g**) Trarg1 S72, a Gsk3 substrate that was regulated by insulin in CHOW and defective in HFD mice. Empirical Bayes moderated two-sided t-tests from the R package "Limma" (version: 3.14) were performed to compare INS to BAS within each diet followed by Benjamini-Hochberg p-value adjustment (* with bracket). * p-value: 0.0444. *: $0.01 < p < 0.05$, **: $0.001 < p < 0.01$, ***: $p < 0.001$. (**h**) Intensity of Gsk3α Y279. n = 12 independent biological replicates.

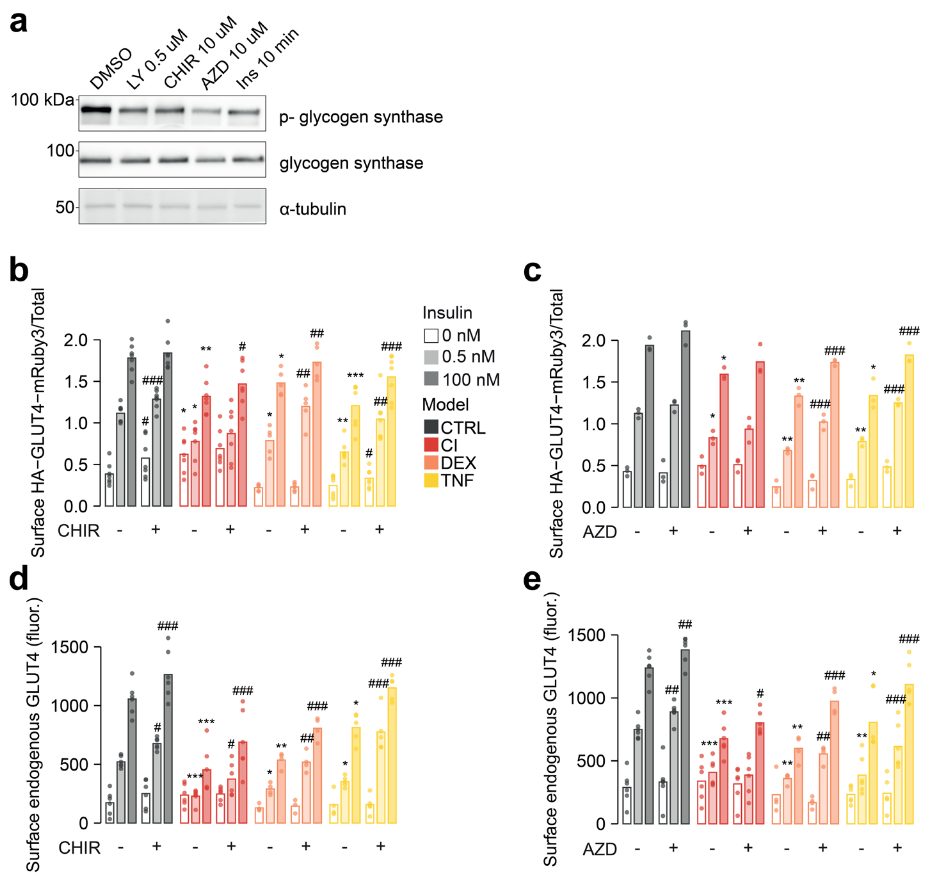

Supplementary Fig. 7 | Pharmacological inhibition of GSK3 (related to Fig. 5)

(**a**) Western blot visualizing phosphorylation of glycogen synthase in 3T3-L1 adipocytes after 90 min incubation in the indicated compounds or 10 min incubation in 100 nM insulin. n = 4 independent biological replicates. (**b-e**) Control and insulin resistant 3T3-L1 adipocytes expressing (**a-b**) HA-GLUT4-mRuby3 or (**c-d**) endogenous GLUT4 were pretreated for 90 min with DMSO or a GSK3 inhibitor (10 µM CHIR99021, "CHIR"; or 10 µM AZD2858, "AZD"), following 20 min insulin stimulation. DMSO-treated conditions in (b) are identical to those presented in Fig. 5a, since the GSK3 inhibitor treatments were performed in the same experiment referencing the same DMSO control. Normalized abundance of HA-GLUT4-mRuby3 or total fluorescence of endogenous GLUT4 at the plasma membrane were compared between control and insulin resistant cells treated with DMSO (*), and between GSK3 inhibitor-treated cells and DMSO-treated cells within each insulin resistance model (#) using two-way ANOVAs and Dunnett's post-hoc tests. The DMSO-treated data in (a) are the same as in **Fig. 5a**. n = 4-6 independent biological replicates. P-values in (b) (left-to-right) *: 0.0164, 0.0191, 0.0080, 0.0359, 0.0190, 0.0011, 0.0003. #: 0.0452, 0.0005, 0.0355, 0.0039, 0.0034, 0.0346, 0.0022, 0.0001. P-values in (c) *: 0.0188, 0.0126, 0.0021, 0.0041, 0.0035, 0.0343. #: < 0.0001, < 0.0001, < 0.0001, < 0.0001. P-values in (d) *: 0.0005, 0.0001, 0.0139, 0.0033, 0.0241, 0.0456. #: 0.0179, 0.0005, 0.0347, < 0.0001, 0.0023, 0.0002, < 0.0001, < 0.0001. P-values in (e) *: 0.0005, 0.0007, 0.0049, 0.0030, 0.0026, 0.0116. #: 0.0062, 0.0047, 0.0196, 0.0012, < 0.0001, < 0.0001, < 0.0001. */#: 0.01 < p < 0.05, **/##: 0.001 < p < 0.01, ***/###: p < 0.001.
