# Supplementary material for: Phosphoproteomics reveals rewiring of the insulin signaling network and multi-nodal defects in insulin resistance
Source: Nat Commun. 2023 Feb 18;14:923. doi: 10.1038/s41467-023-36549-2 (PMC9938909; doi:10.1038/s41467-023-36549-2)
Supplement: Supplementary file 3 — Description of Additional Supplementary Files [file 41467_2023_36549_MOESM3_ESM.pdf]

Description of Additional Supplementary Files

Supplementary Data 1: Proteomic analysis of insulin resistant 3T3-L1 adipocytes
(Page 1 "quantification") Normalized LFQ intensities for proteins that passed filtering for further analysis. (Page 2 "analysis") Statistical analysis of proteome data including models that are changed compared to CTRL.

Supplementary Data 2: Proteomic enrichment analysis
STRING clusters of proteins changed in insulin resistance models. Overrepresentation analysis was performed using KEGG and Reactome pathways on clusters containing 10 or more proteins. (Page 1 "upregulated_clusters") Clusters containing proteins upregulated in 2 or more models compared to CTRL. (Page 2 "downregulated_clusters") Clusters containing proteins downregulated in 2 or more models compared to CTRL.

Supplementary Data 3: Phosphoproteomic analysis of insulin resistant 3T3-L1 adipocytes
(Page 1 "quantification") Normalized LFQ intensities for phosphopeptides that passed filtering for further analysis. (Page 2 "analysis") Statistical analysis of phosphoproteome data including phosphopeptides that are regulated by insulin in control cells, phosphopeptides with defective responses in insulin resistance models, and phosphopeptides with emergent responses in models.

Supplementary Data 4: Canonical insulin signaling proteins
Gene names for proteins that were considered part of the canonical insulin signaling network.

Supplementary Data 5: Phosphoproteomic analysis of 3T3-L1 adipocytes treated with a GSK3 inhibitor
(Page 1 "quantification") Normalized LFQ intensities for phosphopeptides that passed filtering for further analysis. (Page 2 "analysis") Statistical analysis of phosphoproteome data and GSK3 motif analysis. The 290 putative substrate phosphopeptides are identified with the column "Putative substrate".

Supplementary Data 6: Phosphoproteomic analysis of insulin resistant adipose tissue
(Page 1 "quantification") Normalized LFQ intensities for phosphopeptides that passed filtering for further analysis. (Page 2 "analysis") Statistical analysis of phosphoproteome data including phosphopeptides that are regulated by insulin within each diet, phosphopeptides with defective responses in HFD mice, and phosphopeptides with emergent responses in HFD mice.
