# Supplementary material for: Phosphoproteomics reveals rewiring of the insulin signaling network and multi-nodal defects in insulin resistance
Source: Nat Commun. 2023 Feb 18;14:923. doi: 10.1038/s41467-023-36549-2 (PMC9938909; doi:10.1038/s41467-023-36549-2)
Supplement: Supplementary file 10 — Reporting Summary [file 41467_2023_36549_MOESM10_ESM.pdf]

nature portfolio

Corresponding author(s): Sean Humphrey, David James

Last updated by author(s): Jan 17, 2023

Reporting Summary

Nature Portfolio wishes to improve the reproducibility of the work that we publish. This form provides structure for consistency and transparency in reporting. For further information on Nature Portfolio policies, see our Editorial Policies and the Editorial Policy Checklist.

Statistics

For all statistical analyses, confirm that the following items are present in the figure legend, table legend, main text, or Methods section.

n/a	Confirmed	
☐	☒	The exact sample size (*n*) for each experimental group/condition, given as a discrete number and unit of measurement
☐	☒	A statement on whether measurements were taken from distinct samples or whether the same sample was measured repeatedly
☐	☒	The statistical test(s) used AND whether they are one- or two-sided<br>*Only common tests should be described solely by name; describe more complex techniques in the Methods section.*
☒	☐	A description of all covariates tested
☐	☒	A description of any assumptions or corrections, such as tests of normality and adjustment for multiple comparisons
☐	☒	A full description of the statistical parameters including central tendency (e.g. means) or other basic estimates (e.g. regression coefficient) AND variation (e.g. standard deviation) or associated estimates of uncertainty (e.g. confidence intervals)
☐	☒	For null hypothesis testing, the test statistic (e.g. *F*, *t*, *r*) with confidence intervals, effect sizes, degrees of freedom and *P* value noted<br>*Give P values as exact values whenever suitable.*
☒	☐	For Bayesian analysis, information on the choice of priors and Markov chain Monte Carlo settings
☒	☐	For hierarchical and complex designs, identification of the appropriate level for tests and full reporting of outcomes
☐	☒	Estimates of effect sizes (e.g. Cohen's *d*, Pearson's *r*), indicating how they were calculated

Our web collection on statistics for biologists contains articles on many of the points above.

Software and code

Policy information about availability of computer code

Data collection	N/A
Data analysis	DDA: RAW mass spectrometry data was analysed using MaxQuant (vl.6.0.9, vl.6.1.0 and vl.6.17.0 for the adipose tissue phosphoproteomes, 3T3-Ll proteomes, and GSK3i phosphoproteomes respectively). DIA: RAW mass spectrometry data was analysed using Spectronaut (vl4.ll.210528.47784).<br><br>Most analysis was performed in the R programming environment using RStudio (R version: 4.0.3, RStudio version: 1.3.1093-1). Statistical analysis was performed using base R functions as well as the packages "multcomp" (v 1.4-16, Dunnett's post-hoc tests), "limma" (v 3.14, empirical Bayes moderated t-tests, gene set tests), "ksea" (v 0.12, kinase substrate enrichment analysis), and "org.Mm.eg.db" (v 3.15.0, GO enrichment analysis). Visualisations were produced using the R package "ggplot2" (v 3.4.0). Additional statistical analysis was performed in GraphPad Prism (version 9.3.1). Functional protein-protein interaction networks were constructed and analyzed using the STRING web app (https:/ /string-db.org/, version 11.0b). Analysis methods are described in detail in the Methods section.<br><br>Images generated in GLUT4 translocation assays were analyzed using Harmony phenoLOGIC Software (v4.9; Perkin Elmer).

For manuscripts utilizing custom algorithms or software that are central to the research but not yet described in published literature, software must be made available to editors and reviewers. We strongly encourage code deposition in a community repository (e.g. GitHub). See the Nature Portfolio guidelines for submitting code & software for further information.

Data

Policy information about availability of data

All manuscripts must include a data availability statement. This statement should provide the following information, where applicable:

- Accession codes, unique identifiers, or web links for publicly available datasets
- A description of any restrictions on data availability
- For clinical datasets or third party data, please ensure that the statement adheres to our policy

All raw and MaxQuant processed phosphoproteomics data have been deposited in the PRIDE proteomeXchange repository (https://www.ebi.ac.uk/pride/login) and are accessible with the accession PXD032913. Processed data are available as supplementary tables and can be explored online at www.adipocyteatlas.org.
UniProt releases: adipose tissue phosphoproteome (July 2017), 3T3-L1 proteome and GSK3i phosphoproteome (December 2019), 3T3-L1 phosphoproteome (January 2021).

Human research participants

Policy information about studies involving human research participants and Sex and Gender in Research.

Reporting on sex and gender	No human participants were involved in the study.
Population characteristics	No human participants were involved in the study.
Recruitment	No human participants were involved in the study.
Ethics oversight	No human participants were involved in the study.

Note that full information on the approval of the study protocol must also be provided in the manuscript.

Field-specific reporting

Please select the one below that is the best fit for your research. If you are not sure, read the appropriate sections before making your selection.

☒ Life sciences ☐ Behavioural & social sciences ☐ Ecological, evolutionary & environmental sciences

For a reference copy of the document with all sections, see nature.com/documents/nr-reporting-summary-flat.pdf

Life sciences study design

All studies must disclose on these points even when the disclosure is negative.

Sample size	Sample size was based off our previously published study of insulin signalling in 3T3-Ll adipocyte cells (Humphrey et al. 2013).
Data exclusions	*Describe any data exclusions. If no data were excluded from the analyses, state so OR if data were excluded, describe the exclusions and the rationale behind them, indicating whether exclusion criteria were pre-established.*
Replication	All replicates (n's, denoted in each Figure legend, and plotted as individual data points on relevant graphs) were independent biological replicates. All replication experiments were successful.
Randomization	Mouse studies were randomized (non-blinded). Liters of mice (comprising n = 6 animals) were allocated to one of three diet regimes (CHOW, HFD, REVERSAL) randomly. After diet completion, litter mates were randomly assigned to one of the two treatment conditions (insulin OR vehicle). Treatments were randomized to dishes/wells for all cell culture studies.
Blinding	Blinding was not performed for animal studies as it was visually obvious which animals were CHOW or HFD treated due to size differences in both the animals and the volume of fat tissue recovered from each animal.

Reporting for specific materials, systems and methods

We require information from authors about some types of materials, experimental systems and methods used in many studies. Here, indicate whether each material, system or method listed is relevant to your study. If you are not sure if a list item applies to your research, read the appropriate section before selecting a response.

Materials & experimental systems

n/a	Involved in the study
☐	☒ Antibodies
☐	☒ Eukaryotic cell lines
☒	☐ Palaeontology and archaeology
☐	☒ Animals and other organisms
☒	☐ Clinical data
☒	☐ Dual use research of concern

Methods

n/a	Involved in the study
☒	☐ ChIP-seq
☒	☐ Flow cytometry
☒	☐ MRI-based neuroimaging

Antibodies

Antibodies used

Primary antibodies:
pGsk3 S21/S9: Supplier name - Cell Signaling Technology, Catalogue number - 9331S, Lot number - 14, ,Dilution - 1/1000.
Gsk3b total: Supplier name - Cell Signaling Technology, Catalogue number - 9315, Clone name - 27C10, Lot number - 5, Dilution - 1/1000.
14-3-3 total: Supplier name - Santa Cruz Biotechnology, Catalogue number - sc-629, Clone name - K-19, Lot number - A2915, Dilution - 1/5000.
a-tubulin: Supplier name - Sigma, Catalogue number – T9026, Clone name – DM1A, Lot number - 0000164523, Dilution 1/1000.
pGlycogen Synthase (Ser641): Supplier name – Cell Signaling, Catalogue number - 47043, Clone name – D4H1B, Lot number - 1, Dilution 1/1000.
glycogen synthase total: Supplier name – Cell Signaling, Catalogue number - 3886, Clone name – 15B1, Lot number - 3, Dilution 1/1000.
pHDAC4 (Ser246)/pHDAC5 (Ser259)/pHDAC7 (Ser155): Supplier name – Cell Signaling, Catalogue number - 3443, Clone name – D27B5, Lot number - 4, Dilution 1/500.
Endogenous GLUT4 (imaging assays): Supplier name - Integral, Catalogue number – NA (Obtained via MTA), Clone name – LM048, Lot number – 07102019_A01, Dilution 1/1000.
HA (imaging assays): Supplier name - Biolegend, Catalogue number - 901515, Clone name – 16B12, Lot number – B294011, Dilution - 1/500.

Secondary antibodies:
For HA assay – 1/500 - Alexa Fluor 488 goat anti-mouse IgG (H+L), Invitrogen, #A11001, Lot - 2284614.
For endo GLUT4 assay – 1/500 – Alexa Fluor 488 goat anti-human IgG (H+L), Invitrogen, #A11013, Lot - 2273669.
For Western (rabbit) – 1/5000 – goat anti-rabbit IgG (H+L) horseradish peroxidase conjugate , Invitrogen, #G21234, Lot - 2321833.
For Western (mouse) – 1/5000 – Goat anti-mouse IgG (H+L) Alexa Fluor Plus 647 highly cross-adsorbed, Invitrogen, #A32728, Lot – UB275580.
For Gsk3b and 14-3-3: 1/10000 - Donkey anti-rabbit IgG IRDye 800RD, LI-COR, #925-32213
For pGsk3: 1/5000 - Donkey anti-rabbit IgG (H + L) Horseradish peroxidase, Jackson ImmunoResearch, #711-035-152

Validation

pGsk3 S21/S9: validated by manufacturer (CST) for Western Blotting (WB) applications and used in 496 publications (https://www.cellsignal.com/products/primary-antibodies/phospho-gsk-3a-b-ser21-9-antibody/9331).
Gsk3b total: validated by manufacturer (CST) for WB, IP and IHC applications and used in 1128 publications (https://www.cellsignal.com/products/primary-antibodies/gsk-3b-27c10-rabbit-mab/9315).
14-3-3 total: validated by manufacturer (SC) for WB applications and used in 156 publications (https://www.scbt.com/p/pan-14-3-3-antibody-k-19).
a-tubulin: validated by manufacturer (Sigma) for WB, IF and IHC applications (https://www.sigmaaldrich.com/AU/en/product/sigma/t9026).
pGlycogen Synthase (Ser641): validated by manufacturer (CST) for WB, IP and IHC applications (https://www.cellsignal.com/products/primary-antibodies/phospho-glycogen-synthase-ser641-d4h1b-xp-rabbit-mab/47043).
glycogen synthase total: validated by manufacturer (CST) for WB, IP and IHC applications and used in 95 publications (https://www.cellsignal.com/products/primary-antibodies/glycogen-synthase-15b1-rabbit-mab/3886).
pHDAC4 (Ser246)/pHDAC5 (Ser259)/pHDAC7 (Ser155): validated by manufacturer (CST) for WB applications.
Endogenous GLUT4 (imaging assays): extensively validated by us in mouse cells (https://www.life-science-alliance.org/content/6/1/e202201585). Original description of the antibodies - https://pubmed.ncbi.nlm.nih.gov/29769329/.
HA (imaging assays): validated by manufacturer (Biolegend) for WB and IF - CHO and HeLa cells expressing HA-tagged protein.

Eukaryotic cell lines

Policy information about cell lines and Sex and Gender in Research

Cell line source(s)

3T3-L1 fibroblasts were obtained from Howard Green (Harvard Medical School, Boston, MA).

Authentication

3T3-L1's were obtained from the original creator of the 3T3-L1 (Green) cell line. We have confirmed their identity both functionally (i.e., differentiation into functional adipocytes at close to 100% efficiency), and performed RNAseq, MS-proteomics, and MS-phosphoproteomics, each confirming the identity of the cells as mouse adipocytes.

Mycoplasma contamination

Cells were routinely tested for mycoplasma and returned a negative result.

Commonly misidentified lines (See ICLAC register)	No commonly misidentified cell lines were used.

Animals and other research organisms

Policy information about studies involving animals; ARRIVE guidelines recommended for reporting animal research, and Sex and Gender in Research

Laboratory animals	8-week-old male C57BL/6 mice were purchased from Australian BioResources (Moss Vale, Australia), and commenced the experimental regime at 10 weeks of age. Mice were housed at the Charles Perkins Centre Animal Facility, with 4-6 animals per cage at 22°C and 44-46% humidity under 12-h light - 12-h dark cycle settings and free access to food and water. All experiments were carried out with the approval of the University of Sydney Animal Ethics Committee (2014/694), following guidelines issued by the National Health and Medical Research Council of Australia. All studies used at least 24 mice per treatment group (n=12 saline treated, n=12 insulin-stimulated), and studies were performed over 4 separate days spanning two weeks.
Wild animals	No wild animals were used in the study.
Reporting on sex	Only male mice were studied.
Field-collected samples	No field collected samples were used in the study.
Ethics oversight	All experiments were carried out with the approval of the University of Sydney Animal Ethics Committee (2014/694), following guidelines issued by the National Health and Medical Research Council of Australia.

Note that full information on the approval of the study protocol must also be provided in the manuscript.
